# *duper* is a null mutation of Cryptochrome 1 in Syrian hamsters

**DOI:** 10.1073/pnas.2123560119

**Published:** 2022-04-26

**Authors:** Yin Yeng Lee, Sibel Cal-Kayitmazbatir, Lauren J. Francey, Michael Seifu Bahiru, Katharina E. Hayer, Gang Wu, Molly J. Zeller, Robyn Roberts, James Speers, Justin Koshalek, Mark E. Berres, Eric L. Bittman, John B. Hogenesch

**Affiliations:** ^a^Divisions of Human Genetics and Immunobiology, Department of Pediatrics, Cincinnati Children's Hospital Medical Center, Cincinnati, OH 45229;; ^b^Department of Pharmacology and Systems Physiology, University of Cincinnati College of Medicine, Cincinnati, OH 45229;; ^c^Department of Biology, University of Massachusetts Amherst, Amherst, MA 01003;; ^d^Program in Neuroscience & Behavior, University of Massachusetts Amherst, Amherst, MA 01003;; ^e^Institute for Translational Medicine and Therapeutics, University of Pennsylvania, Philadelphia, PA 19104;; ^f^University of Wisconsin Biotechnology Center, University of Wisconsin–Madison, Madison, WI 53706

**Keywords:** fast homozygosity mappings, Cry1-null, short circadian period length, Syrian hamster genome

## Abstract

The *duper* mutation is a recessive mutation that shortens the period length of the circadian rhythm in Syrian hamsters. These animals show a large phase shift when responding to light pulses. Limited genetic resources for the Syrian hamster (*Mesocricetus auratus*) presented a major obstacle to cloning *duper*. This caused the *duper* mutation to remain unknown for over a decade. In this study, we did a de novo genome assembly of Syrian hamsters with long-read sequencing data from two different platforms, Pacific Biosciences and Oxford Nanopore Technologies. Using two distinct ecotypes and a fast homozygosity mapping strategy, we identified *duper* as an early nonsense allele of Cryptochrome 1 (*Cry1*) leading to a short, unstable protein. CRY1 is known as a highly conserved component of the repressive limb of the core circadian clock. The genome assembly and other genomic datasets generated in this study will facilitate the use of the Syrian hamster in biomedical research.

## Significance

We successfully identified the *duper* allele as a null mutation of Cryptochrome 1 in Syrian hamsters. Here, we have shown the use of fast homozygosity mapping as an effective approach to identify causal mutations in mammals, despite lacking chromosomal genome information. In the course of this work, we improved the draft Syrian hamster genome and generated datasets necessary to exploit Syrian hamsters as a modern genetic research model. The unique physiological features of Syrian hamsters make them a desirable model to investigate human diseases, including circadian disorders, cancer, heart function, metabolism, and infectious diseases (e.g., severe acute respiratory syndrome coronavirus 2).

Biological clocks coordinate the timing of biochemical, physiological, and behavioral processes. In mammals, the circadian clock is regulated by a transcriptional-translational negative feedback loop (TTFL) (1). This TTFL drives circadian oscillations of many other clock-regulated genes and results in a gene network that oscillates with a 24-h cycle. Disruption of circadian rhythms is associated with many diseases, including metabolic syndrome, susceptibility to infections, and cancers (2).

*tau* was one of the first mammalian period length mutants identified (3). This allele shortens the free running period of locomotor activity under constant dark conditions (τ) to ∼22 h in heterozygote and 20 h in homozygote (“supershort”) animals. *tau* was identified as a gain of function mutation in a key clock protein kinase, casein kinase 1 epsilon (CKIε), which results in hyperphosphorylation and accelerated degradation of the PER2 protein (4–6). As a consequence, the repressive phase of the TTFL is abbreviated, shortening the period length of locomotor activity.

In prior work, a spontaneous mutation (*duper*) was identified on the homozygous *tau* background (7). These animals had a τ of approximately 18 h and a greatly expanded range of entrainment. To isolate *duper* from the *tau* locus, these animals were backcrossed to the Lakeview (LVG) ecotype. The free running period of locomotor activity rhythms for homozygous *duper* animals was 22.8 h. *duper* heterozygotes had a normal τ, suggesting *duper* is a recessive mutation (7). In contrast to *tau* mutants, *duper* hamsters maintain a high amplitude phase response curve (8, 9).

The limited genetic resources available for *Mesocricetus auratus* (Syrian or Golden hamsters) presented a major obstacle to cloning *duper*. This caused the *duper* mutation to remain unknown for over a decade. In 2000, the *tau* mutation was identified using genetically directed representational difference analysis (GDRDA) (4, 10). GDRDA identifies the polymorphic DNA markers that are tightly linked to a monogenic trait through comparison of “tester” and “driver” pools. GDRDA is a powerful method because it does not need prior knowledge of the chromosomal location of that trait or the availability of genetic maps for the organism. However, GDRDA is a complex and arduous task, especially for a recessive mutation (11).

Fast homozygosity mapping (FHM), a mapping-by-sequencing approach, has been widely applied to screen for recessive mutations (including complex traits) in zebrafish and plants using whole-genome sequencing (12–14). To apply FHM, individuals with the mutation are first crossed to an individual with a distant genetic background. The principle behind FHM is that a fraction of the genome of offspring with the phenotype would be expected to be homozygous, and linked to the phenotypic parental genetic background. Pooling DNA from a large cohort of phenotypic offspring will help to further segregate the mutations and localize the causal variants.

A good genome assembly for the species is needed for the FHM approach. Efforts to build a reference genome for the Syrian hamster started recently (15, 16) because this species is an excellent model to study many types of cancers, viruses, and heart disease. The contiguity of the genome assembly is important to map the allele using FHM. We used Pacific Biosciences (PacBio) and Oxford Nanopore Technologies (ONT) long-read sequencing to reassemble the Syrian hamster genome and improve its contiguity. Using the newly assembled reference genome, we successfully applied FHM to identify a ∼10.6 Mbp genomic region linked to *duper*. Further analysis revealed that *duper* is a Cryptochrome 1 (CRY1) null mutation of Syrian hamsters, caused by a frameshift deletion in exon 4 that leads to an early stop codon. CRY1 is known as a highly conserved component of the core circadian clock (17). Our study identified *duper* as a CRY1-null Syrian hamster. Tools and datasets generated through the course of this work will help to facilitate the use of this species as a modern genetic animal model to study circadian, metabolic, infectious, and cardiovascular diseases.

## Results

### *duper* Allele Isolation and Characterization.

**Fig. 1.**
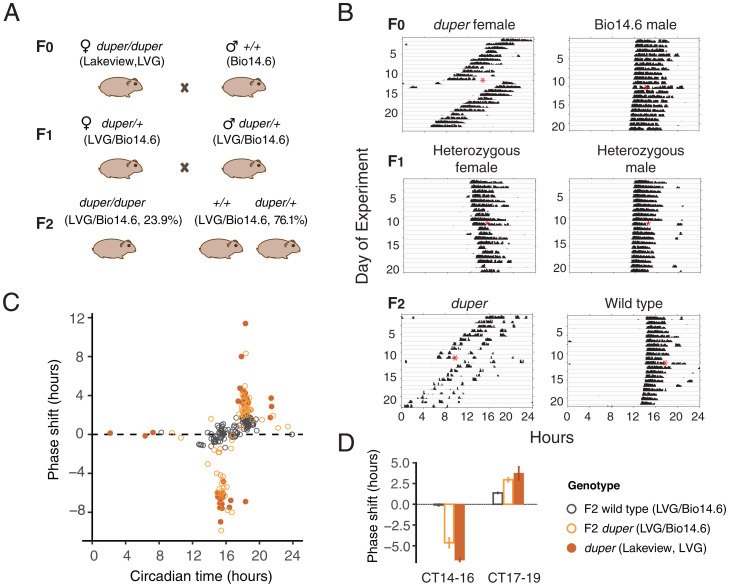
Isolation and characterization of *duper* allele in Syrian hamsters. (*A*) Mating scheme for fast homozygosity mappings. (*B*) Actograms of *duper* and wild type hamsters. Red asterisks indicate when a 15′ light pulse was given. (*C*) Phase response curve established the response to 15′ light pulses. F2 wild-type (LVG/Bio14.6), F2 *duper* (LVG/Bio14.6), and *duper* (LVG) are shown as gray open circles, orange open circles, and filled orange points, respectively. (*D*) Bar graph show phase delays and advances in response to 15′ light pulse presented at CT14-16 and CT17-19, respectively (mean ± SEM).

*duper* arose on the homozygous *tau* background. A female *duper* hamster (LVG ecotype) was crossed with a wild-type male hamster (Bio14.6 ecotype) to move the *duper* allele to a different genetic background (Fig. 1*A*). The paternal line arose as a spontaneous mutation and was maintained for over 50 y by BioBreeders (Fitchburg, MA) as a separate lineage for studies of cardiomyopathy and muscular dystrophy resulting from deletion of d-sarcoglycan (18). Similar to previous observations (7), the *duper* phenotype transmitted as a recessive mutation in this cross. The τ of the F1 heterozygotes was ∼24 h, similar to the period of wild-type hamsters (Fig. 1*B*). We inbred the F1 heterozygotes and generated F2 animals (*n* = 197). F2 hamsters were phenotypically separated using the period length of their free-running locomotor activity. In total, 47(23.9%) hamsters showed the short τ (*duper*) phenotype, with a mean period of 22.73 ± 0.1 h (mean ± SEM). The remaining F2 hamsters had a mean period of 24.04 ± 0.01 h.

To map the phase response of the F2 hamsters, animals maintained in DD were exposed to a 15-min (15′) light pulse in the early or late subjective night. Hamsters with the *duper* mutation showed a mean phase delay of 4.64 ± 0.57 h when given a light pulse at CT15.29 ± 0.08 h. When these hamsters were given a 15′ light pulse at CT18.27 ± 0.06 h, they showed a mean phase advance of 2.95 ± 0.24 h. In contrast, wild-type F2 hamsters given light pulses at CT15.17 ± 0.08 h showed a phase delay of 0.08 ± 0.10 h. Light pulses at CT18.36 ± 0.05 h elicited advances of 1.36 ± 0.07 h (Fig. 1 *C* and *D*). Thus, responses of the F2 *duper* hamsters to 15′ light pulses were comparable to those of the F0 *duper* hamsters of the LVG background. The *duper* phenotype transmits without apparent modification on the Bio14.6 background.

### Reassembly of the Syrian Hamster Reference Genome.

FHM identifies genetic regions that are homozygous and conserved in the F2 offspring that match to the phenotypic parental genetic background. The contiguity of the assembly is important to successfully map the region, because longer scaffolds of the reference genome allow us to identify genomic regions linked to the phenotype with higher confidence. The first reference genome of the Syrian hamster (MesAur1.0) was published in 2013 using short sequencing reads. This genome assembly has 21,483 scaffolds and 237,699 contigs, with N50 of 12,753,307 and 22,512, respectively. This assembly is fragmented, with many gaps and missing information at the low complexity region. To improve the structure of the reference genome, we used PacBio (Sequel System) and ONT (GridION) to generate long-reads and then reassembled the reference genome of the Syrian hamster (Table 1). Our reference genome greatly reduced the total contigs and scaffolds to 3,056, with a N50 of 26,783,296. Benchmarking Universal Single-Copy Orthologs (BUSCO) assessments with the 9,226 mammalian markers (19), the estimated completeness of this assembly was 88.2%.

**Table 1.** Statistics of the de novo genome assembly

Parameters	Measurements
Contigs and scaffolds	3,056
Total length (bp)	2,500,764,195
N50/N90 (bp)	26,783,296/2,215,234
GC%	41.8%
BUSCO	88.2%

### Fast Homozygosity Mapping Identifies the *duper* Allele in Syrian Hamsters.

Genomic DNA was isolated from a *duper* (LVG) animal, a wild-type (Bio14.6) animal, and a pool of 45 phenotypically selected F2 homozygous *duper* mutants (LVG/Bio14.6). Whole-genome sequencing was performed using paired-end Illumina sequencing. Read alignment and variant calling were done following GATK best practices (20). Variants with at least 5× total coverage were used for further analysis.

FHM used unique homozygous single nucleotide polymorphisms (SNPs) from either parent as markers to identify genomic regions in the F2 phenotypic offspring that linked to the parental genetic background. We identified 215,699 and 801,403 unique homozygous SNPs in wild type (Bio14.6) and *duper* (LVG), respectively (Table 2). The newly assembled reference genome was generated from a Syrian hamster from the LVG ecotype. Its genetic distance is nearer to the *duper* (LVG) background in comparison to wild type (Bio14.6). Therefore, the number of SNPs called in wild type (Bio14.6) is 3-fold more than *duper*. The curated markers have a higher density of Bio14.6 SNPs such that it will dominate the homozygosity score calculation.

**Table 2.** Number of unique homozygous SNPs in the two Syrian hamster ecotypes

Ecotypes	No. of unique homozygous SNPs
Lakeview (F0 *duper/duper*)	215,699
Bio14.6 (F0 +/+)	801,403

**Fig. 2.**
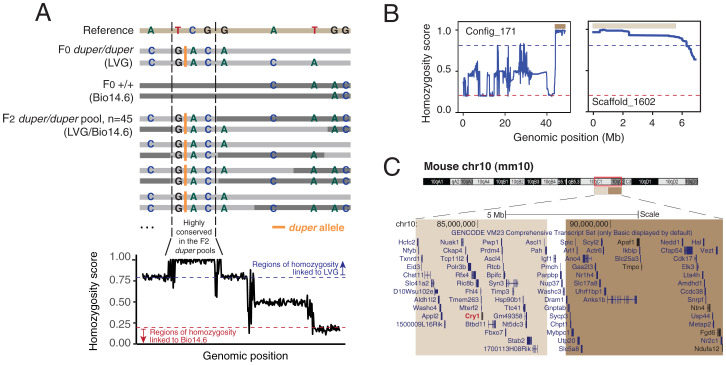
Identification of genomic regions linked to *duper*. (*A*) Schematic diagram representing the principle underlying homozygosity mapping. (*B*) Fast homozygosity mapping identified two regions that have high homozygosity scores and was linked to the *duper* LVG ecotype (brown and light brown). (*C*) Both LVG-linked homozygous regions mapped to 10qC1-C2 in the mouse genome (mm10). *Cry1* (red) is the only known clock gene of the TTFL loop found in this region.

We modified the scoring approach to reduce the impact of imbalanced SNPs from the parental background. Instead, we scanned through all SNPs (both homozygous and heterozygous) from F2 *duper* pools with coverage larger or equal to 5 (*n* = 1,232,905) and assigned different weights to these SNPs. Detailed scoring criteria is described in the *Material and Methods*. We calculated the average homozygosity score in a rolling 100 SNPs window. Regions with homozygosity score >0.9 and <0.1 suggested the region is highly conserved in the F2 *duper* pools and were linked to LVG and Bio14.6, respectively (Fig. 2*A*).

Using this approach, we identified two highly conserved homozygous regions (∼4.8 and 5.8 Mbp, respectively) in the F2 *duper* pools inherited from the LVG background (Fig. 2*B*). The Syrian hamster reference genome still lacks chromosomal-level information. For that reason, we used the Basic Local Alignment Search Tool (BLAST) to map these two regions to the mouse genome. Interestingly, both regions mapped near each other on mouse chromosome 10 (build mm10) at 10qC1-C2 (Fig. 2*C*). Given these findings, we reasoned that the *duper* locus resides in this ∼10.6 Mbp genomic region.

**Fig. 3.**
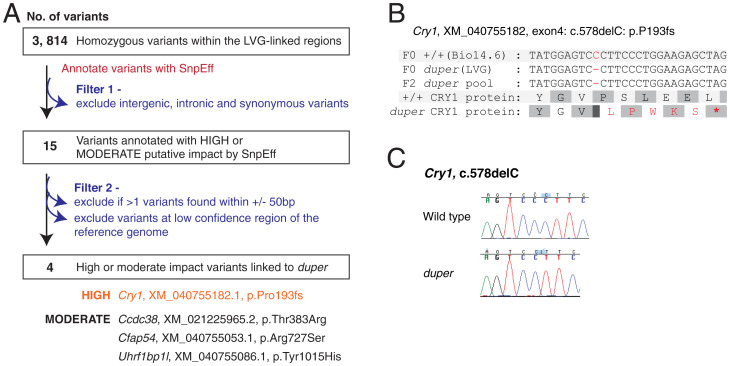
Identification of the *duper* allele. (*A*) Filtering process to identify candidate variants of *duper* alleles. One high-putative impact variant (*Cry1*) and 3 moderate-putative impact variants remained after the filtering process. (*B*) The p.Pro193fs mutation in *Cry1* was predicted to cause an early stop codon in the CRY1 protein in *duper*. (C) Sanger sequencing validation of the 1bp deletion in *duper*.

We restricted our search for candidates to unique *duper* variants (both SNPs and INDELs). These candidates represent variants found in F0 and F2 *duper* but not in our mapping ecotype, Bio14.6. We annotated these variants with SnpEff (21) and filtered out intergenic, intronic, and synonymous variants (Fig. 3*A*). This identified 15 variants to have high- or moderate-putative impact. We manually screened these variants and filtered out those from low confidence genomic regions. After applying these filters, four candidate variants remained (Fig. 3*A*). Among these candidate variants, *Cry1* is the only gene known to encode a core clock protein (1). A 1-bp frameshift deletion in exon 4 of *Cry1* (c.578delC; p.P193fs) was predicted to cause an early stop codon in the CRY1 protein (Fig. 3*B*). In *duper*, the 1-bp deletion was validated using Sanger sequencing. This mutation, associated with the *duper* phenotype, was not found in the wild-type hamsters (Fig. 3*C*).

### CRY1 Protein Is Not Expressed in *duper* Hamsters.

**Fig. 4.**
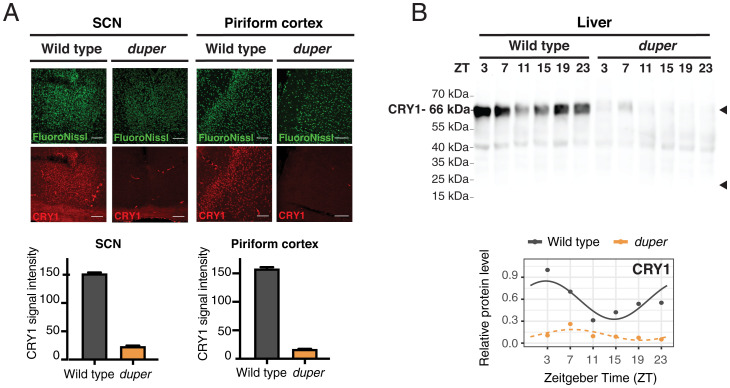
Validation of CRY1-null in the *duper* hamsters. (*A*) Representative immunocytochemistry images of *duper* and wild-type hamster brain sections from ZT12 containing SCN and piriform cortex. (Scale bar: 100 μm.) Bar plot indicates the quantifications of CRY1 signal intensity in SCN and piriform cortex, respectively. (*B*) Western blotting was performed on total protein extracts with N-terminal CRY1 antibody in wild-type and *duper* hamsters from liver tissues collected at ZT3, ZT7, ZT11, ZT15, ZT19, and ZT23. Normalized levels of the full size CRY1 protein (66.3 kDa) are shown in the plot. Significance of the rhythmicity test was performed using CircaCompare and represented with solid dark gray line (*P* = 0.075) and dotted orange line (*P* = 0.148).

The *duper* allele is predicted to cause an early stop codon in *Cry1* in Syrian hamsters. We performed immunocytochemistry using an antibody directed at the N-terminal region of CRY1 to determine whether protein is expressed in brain regions of Syrian hamsters. We observed a profound reduction of CRY1 staining in *duper* relative to wild-type hamsters at ZT12 in both suprachiasmatic nucleus (SCN) and piriform cortex (Fig. 4*A*). Next, we performed Western blot analysis using an N-terminal CRY1 antibody on whole cell lysates of liver tissues collected at ZT3, ZT7, ZT11, ZT15, ZT19, and ZT23. The predicted truncated protein is 22.5 kDa, while the full size CRY1 is 66.3 kDa. We detected full size CRY1 proteins in wild-type animals, but detected neither the full size nor the truncated CRY1 protein in *duper* animals (Fig. 4*B*). Taken together, these data suggest that *duper* is indeed a CRY1-null in hamsters.

Commercially available hamster-specific antibodies are very limited. In addition to CRY1, we were able to detect three other core clock proteins (PER1, PER3, and CRY2) in hamsters using these antibodies (*SI Appendix*, Fig. S1 and Table S1). While total protein levels do not show a significant difference, we observed an ∼6 h phase advance in PER3 abundance in the liver of *duper* animals (CircaCompare, *P* = 0.004). No significant changes were observed between *duper* and wild type in the time course of expression of PER1 or CRY2 in the liver.

We also compared genes expressed in three tissues (cortex, SCN, and liver, RNA sequencing [RNA-seq]) between a pool of four *duper* and four wild-type hamsters collected at single time point (CT15). As expected, expression levels of *Cry1* were significantly reduced in *duper* animals (*SI Appendix*, Fig. S2). In accordance with Western blot results, *Per3* expression was reduced in the liver at CT15. However, similar changes were not observed in the SCN and cortex, suggesting the changes of PER3 might be liver specific. No significant changes were observed for other core clock genes at the transcriptional level at CT15.

Additionally, we identified 39 genes differentially expressed in the three tested tissues in *duper* (*SI Appendix*, Fig. S3 and Dataset S1). Several of these genes are not well-characterized in the Syrian hamster genome. We selected the 15 well-annotated differentially expressed genes for pathway enrichment analysis. Five of these genes, including *Nat2*, *Cryl1*, *Alas2*, *Aldh1a3*, and *Gapdh*, are enriched in metabolic pathways (*P* = 0.09). Two of them, *Irf7* and *Isg15*, are enriched in RIG-I-like receptor signaling pathways (*P* = 0.09). Further experiments with time-series tissue collections and more technical replicates would be helpful to elaborate transcriptomic changes of *duper*, a CRY1-null hamster model.

## Discussion

We identified the underlying mutation of *duper* as a frameshift deletion in exon 4 of *Cry1* that generates a premature stop codon, resulting in the absence of CRY1 protein. *Cry1* plays a prominent role in regulating the period length of locomotor activity in mice. Studies in CRY1-null mice found shortening of τ similar to *duper* (22). A large-scale N-ethyl-N-nitrosourea mutagenesis screen in mice identified a mutant termed *part-time* with a short period circadian locomotor activity phenotype. This mutation was mapped and found to be a nonsense mutation at the 3′-exonic region of exon 2 in *Cry1* (23). In humans, the c.1657 + 3A > C allele results in skipping of exon 11 in *CRY1* (CRY1 Δ11) and segregates with delayed sleep-wake phase disorder. In contrast to the loss-of-function CRY1-null, CRY1 Δ11 represents a gain-of-function mutation. Exon 11 plays an important role in the interaction between the CRY1 tail and its photolyase homology region. CRY1 Δ11 results in enhanced interactions with CLOCK:BMAL1 proteins and increased inhibition of transcription for the CLOCK:BMAL1 targeted genes (24, 25)

Other than shortening τ, we also observed a dramatic shortening of the latency to re-entrain upon 8 h or 12 h shifts of the light:dark (L:D) cycle in *duper* hamsters compared to wild-type. Similar responses are observed in Cry1-null mice upon 6 h shifts of the L:D cycle (26). Additionally, *duper* hamsters show a high amplitude phase response curve, with shifts of up to 12 circadian hours in response to a 15′ light pulse (Fig. 1*C*) (8). Such a marked increase in amplitude of the PRC has not been reported in CRY1-null mice (27).

Notable evolutionary differences exist in light entrainment. *Drosophila* cryptochrome is a flavin adenine dinucleotide (FAD)-dependent circadian photoreceptor, whereas mammalian cryptochromes (CRY1 and CRY2) are integral clock components that repress CLOCK/BMAL1-dependent transcription (28). Interestingly, while cryptochromes play a role in light entrainment in flies, in mammals, it functions as the critical repressive component of the TTFL. Recent studies suggest that the FAD pocket of mammalian CRY2, but not CRY1, retains some function and possibly works in a light-dependent manner (29). A mutation in the CRY2 FAD binding domain, p.A260T, was segregated with familial advanced sleep-wake phase disorder. This mutation promotes FBXL3 binding and therefore increased degradation of CRY2. The humanized p.A260T CRY2 transgenic mice show a subtle shortened period and altered sensitivity to photic entrainment (30). This suggests CRYs play an important role in both the period of the circadian clock and light-induced phase shifting in humans. *duper* may provide a unique model for light entrainment studies.

*duper* was segregated from a spontaneous mutation that arose on the homozygous *tau* hamster background. Interestingly, the combination of *duper* and *tau* mutations in hamsters (termed “super *duper*”) showed significant shortening of τ to ∼18 h, which is the shortest circadian period of locomotor activity so far recorded in mammals (9). The interaction between mutations affecting PER and CRY was described in studies of mice carrying both the *tau* and *afterhours* mutations. *tau* mutations lead to gain of function CKIε that hyperphosphorylates PER proteins and destabilizes them, leading to a shortened repressive phase. *Afterhours* is caused by a mutation in FBXL3 that stabilizes CRY proteins and lengthens the period. The circadian effects of Fbxl3*^Afh^* and CKIε*^tau^* were independent and additive (31). This contrasts with the phenotype of the super *duper* hamsters, in which the combination of the CKIε gain-of-function with the effective loss of CRY1 results in an epistatic interaction: the shortening of period in *duper* (~6 h) is more than additive of CKIε (~4 h) and CRY1-null (~1 h). We hypothesize that super *duper* hamsters have hyperphosphorylated PER2 that accelerates nuclear entry of PER:CRY2 complexes to initiate the early repressive phase. In the absence of CRY1, the late repressive phase may be truncated or abbreviated in *duper* animals. Since CRY proteins prevent ubiquitylation of PER2 (32), PER may be further destabilized in the super *duper* hamsters. Further exploration of the mechanism by which *tau* and *duper* mutations interact, not only to shorten period but also to amplify phase resetting, will provide further insights into interactions of components of the TTFL.

Beyond its importance as a repressor of the TTFL in circadian clocks, CRY1 has been found to regulate metabolic oscillations (33, 34) and participate in DNA damage response (35) and cancer (36). Surprisingly, we found no publicly available CRY1-null transcriptomics datasets in any animal models to date. Using our RNA-seq data, we sought to explore the possible functional consequences of CRY1-null and circadian alteration in hamsters at CT15.

RNA expression profiling showed alterations of the RIG-I-like receptor signaling pathway in *duper*, which may link the CRY1-null *duper* animals to the viral sensing response. Both the enriched genes, *Irf7* and *Isg15*, are not rhythmic in any tissues in mice (37), suggesting that the observed changes are not likely due to phase shifts in the animals. *Uba7*, the gene encoding enzyme UBE1L that activates ISG15 protein conjugation (38), was also up-regulated in *duper* animals. We cross-checked changes of these three genes with a published *CRY1* knockdown RNA-seq dataset (GSE144961) (36). Similar to *duper* animals, these three genes are up-regulated in *CRY1* knockdown in human cells, albeit with more subtle changes (*SI Appendix*, Fig. S4). The interplay between the circadian clock and viral infection has been previously reported (39). ARNTL-null mice were found to be more susceptible to viral infections, however, all these studies explored responses to viral infections in arrhythmic animals (40–42). Higher viral replication is observed at ZT0 in comparison to ZT10 (40). This aligns with the rhythmicity of CRY1 protein levels, where CRY1 peaks at ZT0 and reaches a nadir at ZT10. Whether *Cry1* inhibits (directly or indirectly) the RIG-I-like receptor signaling pathway, in particular IRF/ISG transcription, remains to be explored.

A significant decrease in *Nat2* in the SCN and cortex is observed in our transcriptomics data. Although AANAT is thought to be the rate limiting enzyme for melatonin synthesis in the pineal gland (43), changes in *Nat2* expression may contribute to changes in circadian and seasonal responses in *duper* animals.

Our pilot RNA-seq datasets do not conclusively represent CRY1-null transcriptomics profiles in hamsters. First, we only tested a single time point (CT15). Second, we only tested three tissues without replicates. We did stringent analysis and found 15 well-annotated genes altered in all three tissues analyzed (cortex, SCN, and liver), 8 of which are enriched in two specific pathways: RIG-I-like receptor signaling and metabolic pathways. This observation suggests the need for future exploration of *duper*, a CRY1-null model. Increasing temporal resolution and replicates for each tissue will help to better characterize the transcriptomic profiles of the animals.

The utility of Syrian hamsters has declined since the early 2000s, largely due to the lack of genetic and molecular resources available. In the past few years, long-read sequencing has facilitated the effort to build high quality genome assemblies for other mammals (44). In this study, we used long-read sequencing from PacBio and ONT to improve the scaffolding and contiguity of the Syrian hamster genome. Using this assembly and the prior knowledge of the mouse genome, we successfully applied FHM to identify the *duper* locus. In the course of this work, we also sequenced cDNA collected from three hamster ecotypes, including the LVG, Bio14.6, and hamsters descended from a few generations wild caught (*SI Appendix*, Fig. S5) (45). Tools and data generated in this study expand the genetic resources available for Syrian hamsters.

The unique physiological features of the Syrian hamster make it a desirable model to investigate human diseases, including cancer, heart function, metabolism, and infectious diseases (e.g., severe acute respiratory syndrome coronavirus 2) (46, 47). Furthermore, hamsters offer a superior model for study of seasonal and photoperiodic responses (48). Future work to complete and annotate the reference genome for Syrian hamsters will enable its exploitation as a modern genetic model for biomedical research.

## Materials and Methods

### Animal Models.

All Syrian hamsters (*M. auratus*) were born and raised in a 14:10 L:D cycle and allowed ad libitum access to food and water throughout the study. Wild-type hamsters of the Lakeview (LVG) ecotype were obtained from Charles River Laboratories (Wilmington, MA) or bred in our laboratory from that stock. *duper* hamsters used in these studies were derived from the original super *duper* animals crossed to LVG as previously described (7). They descended from animals that were confirmed by restriction digest mapping of their genomic DNA (4, 7) to lack the *tau* mutation and in which τ was ∼23 h. The Bio14.6 hamsters were obtained from BioBreeders (Farmington, MA). All experiments were approved by the IACUC of the University of Massachusetts Amherst (Protocol 2013–0076).

### Light Pulse and Phase Response Curve.

Circadian phenotype was assessed by maintenance in tub cages containing running wheels; locomotor activity rhythms were assessed using Actimetrics software as previously described (8). The phenotype of the parental (F0) Bio14.6 and *duper* hamsters and of their F1 offspring was evaluated by quantification of free running period over the course of at least 10 d in DD and response to 15′ light pulses administered in early to midsubjective night. In order to confirm the phase shifting phenotype of the F2 animals, activity rhythms of 79 hamsters were assessed in DD and phase shifts were quantified after 15′ light pulses at approximately CT15.5 and CT18. To determine whether the *duper* phenotype was transmitted on the Bio14.6 background, we constructed a phase response curve to compare shifts elicited in *dupers* on wild type (LVG) background with the F2 animals. In all cases, the phase shift was determined by linear regression of activity onsets upon establishment of steady state in the 10 d following the light pulse.

### Long-Read Sequencing, De Novo Genome Assembly, and Annotation.

DNA was extracted from whole blood obtained from a female wild-type LVG outbred golden Syrian hamster (*M. auratus*). Genomic DNA libraries were prepared for sequencing on two different long-read platforms, Oxford Nanopore Technologies (GridION; LSK109) and Pacific Biosciences (Sequel System; SMRTbell Express Template Prep Kit 2.0). Prior to sequencing, the Oxford Nanopore library was processed with the Short Read Eliminator XL Kit (Circulomics) following manufacturer’s directions. One single-molecule real-time (SMRT) cell and four MinION flow cells generated the entirety of data used in the assembly of the Syrian Hamster genome. Oxford Nanopore adapters and reads containing evidence of internal adapters were removed with Porechop v0.2.4 (https://github.com/rrwick/Porechop). The remaining reads were then filtered with Filtlong v0.2.0 (https://github.com/rrwick/Filtlong), requiring a minimum read QV of 9 and length of 5 Kb. Flye v2.7 (49) was used to generate a de novo assembly of the filtered nanopore reads requiring a minimum of 8 Kb overlap among reads. Error correction was performed independently with gcpp v2.0.2 (https://github.com/PacificBiosciences/gcpp) using pbmm2 v1.1.0 alignments (https://github.com/PacificBiosciences/pbmm2) of Pacific Biosciences subread data to the draft assembly. The resulting assembly of error-corrected contigs was assessed for completeness using lineage-specific single-copy orthologs (mammalia_odb10) with BUSCO v4.0.6 (19). Liftoff v1.6.1 (50) was used to annotate the draft assembly with BCM_Maur_2.0 (GCF_017639785.1) serving as the reference annotation source. All portions of the workflow were performed by the University of Wisconsin–Madison Biotechnology Center.

### Whole-Genome Sequencing, Genome Alignment, and Variants Calling.

Genome DNA was prepared from F0 male (Bio14.6), F0 female *duper* (Lakeview) hamster and a pool of phenotypically identified F2 *duper* offspring (*n* = 45). Libraries from these three samples were pooled and split equally into three lanes of the flow cells. Whole-genome sequencing was run with 2 × 150 bp sequencing on the Illumina 4000 platform. Sequenced data were aligned to the newly assembled Syrian hamster genome assembly mentioned above, using Burrows-Wheeler Aligner (51) (v0.7.17) with the “bwa mem” command. BAMs from the same samples were merged using samtools (52) (v1.9.0) merge. Duplicate reads were removed and base quality were recalibrated as suggested in the best-practice using GATK (20) (v4.1.2). HaplotypeCaller (53) from GATK were used to call variants in each sample for each individual contig separately. All SNPs/INDELs from the same samples were merged to a vcf file using MergeVcf and filtered with parameter suggested by the best practice guidance of GATK.

### Fast Homozygosity Mapping and Variants Filtration.

We modified the fast homozygosity mapping strategy as described in Voz et al. (12). We scanned through all SNPs from F2 *duper* pools with coverage ≥5 (*n* = 1,232,905) and calculated the average homozygosity score in a rolling 100 SNPs window, using scoring criteria listed below.

I. For all homozygous SNPs from the *duper* pools, skipped if it is a homozygous SNP in both the parental samples. Assigned a score of 1 for unique homozygous SNP in the Lakeview sample, a score of 0 for unique homozygous SNP in the Bio14.6 sample, and a score of 0.5 otherwise.
II. For all heterozygous SNPs from the *duper* pools, we assigned a score of 0.8 for unique homozygous SNP in the Lakeview sample, a score of 0.2 for unique homozygous SNP in the Bio14.6 sample, and a score of 0.5 otherwise.

Windows with homozygosity scores ≥0.9, ≤0.1 were merged to indicate strong linkage to Lakeview or Bio14.6 alleles, respectively.

We restricted the search for candidate mutations in the LVG linked region. SNPs predicted to cause a HIGH or MODERATE putative impact estimated by SnpEff (21) were being screened manually using Integrative Genomics Viewer (54) to exclude potential false-positive variants. Given that this was a drafted assembly, some transcripts, especially those with lower complexity or higher repetitive regions, were mapped with lower confidence. We filtered variant(s) with more than 1 variant found within 50 bp up- or downstream, and variants found in exonic regions with no complete reading frames (e.g., multiple stop codons found in all possible reading frames).

### Sanger Sequencing Validation.

To confirm the candidate mutations in *Cry1*, cDNA was prepared from total RNA extracted from cortex and hypothalamus of wild-type and *duper* hamsters. We prepared cDNA with 500 ng of RNA input using the High Capacity RT kit (Applied Biosystems) manufacturer’s protocol. cDNA was diluted 1:10, and 2 μL were used as template per reaction for the Phusion High-Fidelity DNA Polymerase (NEB) PCR amplification following the manufacturer’s protocol with an annealing temperature of 60 °C. The forward (GAGATGCCAGCAGAGACCA) and reverse (GGCTCGCAAGCAGGGAAT) primers produce a predicted amplicon of 253 bp, which was confirmed by gel electrophoresis. Sanger sequencing was performed at the DNACore (Cincinnati Children’s Hospital) following an enzymatic digest with ExoSAP-IT (Applied Biosystems). Two individual reactions were sequenced with both forward and reverse primers. Sequencher (GeneCodes) was used for sequence alignment and analysis.

### Immunocytochemistry.

Adult male *duper* and wild-type hamsters (*n* = 3) were anesthetized with pentobarbital (80 mg/kg) and transcardially perfused with cold phosphate-buffered saline (100 mL) and 4% paraformaldehyde (300 mL) at ZT12. Brains were postfixed overnight, infiltrated with 20% sucrose in phosphate buffer, and frozen sectioned at 40 um. Sections were denatured in sodium citrate for 30 min at 92 °C. After buffer rinses and blocking in normal serum, sections were incubated with an antibody directed at the N-terminal of CRY1 (Novus; NBP1-69080; 1:100) overnight followed by a 3-h incubation in Cy3-conjugated donkey anti-rabbit (1:350). Before mounting, sections were incubated in Neurotrace FluroNissl 500/525 for 30 min (ThermoFisher, N21480, 1:300). Immunostaining was assessed using a Zeiss 700 confocal microscope as previously described (55).

### Western Blotting.

Liver tissue (30 mg) was processed in RIPA buffer with protease and phosphatase inhibitors (SIGMAFAST Protease Inhibitor Mixture Tablets, EMD Millipore Phosphatase Inhibitor Mixture Set 1 and 2). A dounce homogenizer was used to homogenize the tissues in the RIPA buffer. Homogenized lysates were centrifuged at 15,000 × *g* for 20 min and the clear phase supernatant was collected as the whole cell lysate. We performed a BCA (bicinchoninic acid) assay (Sigma) to determine protein concentrations in the lysates. We mixed the lysates with NuPAGE LDS Sample Buffer (Thermo Fisher) and boiled them at 95 °C for 10 min. SDS-PAGE was used to separate the proteins based on their molecular weight. We used the Trans-Turbo Transfer System (Bio-Rad) to transfer the proteins from SDS-PAGE to the PVDF membrane. After blocking the membranes with 5% bovine serum albumin, we used CRY1-N-terminal binding antibody provided by the Lamia Lab to detect CRY1. PER1 (ab136451), PER3 (ab177482), and CRY2 (ab93802) antibodies were purchased from Abcam. The ChemiDoc MP (Bio-Rad) is used to detect chemiluminescence signals from the blots and ImageLab analysis software (Bio-Rad) was used to determine the band intensities. Stain-Free Imaging Technology by Bio-Rad is used for normalization purposes. Briefly, the trihalo compound in the gel generates fluorescence signals by binding to tryptophan residues. Thus, the total protein amount in the membrane is detected and used to normalize bands in each lane. Relative protein expressions were tested for circadian expression differences using CircaCompare (56).

## Supplementary Material

Supplementary File

Supplementary File

## Data Availability

Genome sequencing data have been uploaded to Sequence Read Archive (accession no. PRJNA754138). Transcriptomics data have been uploaded to Gene Expression Omnibus (accession no. GSE184205). Syrian hamster genome is associated with BioSample (accession no. SAMN26084744). Analysis code for the fast homozygosity mapping is available at GitHub, https://github.com/yyenglee/FHM-duper.
