# Supplementary material for: duper is a null mutation of Cryptochrome 1 in Syrian hamsters
Source: Proc Natl Acad Sci U S A. 2022 Apr 26;119(18):e2123560119. doi: 10.1073/pnas.2123560119 (PMC9170138; doi:10.1073/pnas.2123560119)
Supplement: Supplementary File [file pnas.2123560119.sapp.pdf]

PNAS
www.pnas.org

Supplementary Information for

duper is a null mutation of Cryptochrome 1 in Syrian hamsters

Yin Yeng Lee[1], Sibel Cal-Kayitmazbatir[1], Lauren J. Francey, Michael Seifu Bahiru, Katharina E. Hayer, Gang Wu, Molly J. Zeller, Robyn Roberts, James Speers, Justin Koshalek, Mark E. Berres, Eric L. Bittman, John B. Hogenesch*

[1]These authors contribute equally.

*Corresponding Author. Email: john.hogenesch@cchmc.org

This PDF file includes:

Supplementary method
Figures S1 to S5
Table S1
Legend for Dataset S1

Other supplementary materials for this manuscript include the following:

Dataset S1

Supplementary method

RNA sequencing and differential gene expression analysis. RNA was extracted from liver, cortex and SCN from 4 *duper* (4 female) and 4 wildtype (2 male, 2 female) hamsters collected at CT15 (TRIzol Invitrogen). RNA from cortex and SCN from the same genotypes was pooled together before library constructions. 1 µg of total RNA was used for library preparation with the TruSeq Stranded mRNA Sample Preparation Kit (Illumina). Libraries from twelves samples (2 cortex, 2 SCN and 8 livers) were pooled and each library was subjected to 2×100bp sequencing in two lanes on a HiSeq 2000 sequencer (Illumina). Sequencing reads were aligned to the hamster reference genome (MesAur2.0) using Kallisto(1). Estimated counts for transcripts from the same genes were added up using the tx2gene function integrated in tximport. DESeq2(2) was used to call the differentially expressed genes between duper and wildtype, adjusted for tissue-specific differences. Pathway enrichment analysis was done through DAVID web tools(3).

Differential expression analysis for RNA-seq data GSE144961. Read counts for dataset GSE144961 were downloaded from GEO. DESeq2(2) was used to call the differentially expressed genes between control(shCon) and CRY1 knockdown(shCRY1) cells.

RNA sequencing and variants calling. RNA sequencing data were processed as suggested by the best-practice workflows using GATK. Reads from RNA sequencing data were aligned to the newly assembled Syrian hamster genome assembly mentioned above, using STAR aligner v2.7.4 (4) two-pass mode. Duplicate reads were removed and SplitNCigarReads were run to split reads with Ns in Cigar string. HaplotypeCaller from GATK v4.1.2 were used to call variants in each sample. We filtered variants with the same parameters “QD<2.0, FS>30.0” and adjusted minimum coverage (DP) based on sequencing depth of each sample.

SI References

1. Nicolas L Bray, Harold Pimentel, Páll Melsted and Lior Pachter, Near-optimal probabilistic RNA-seq quantification, *Nat. Biotechnol* **34**, 525–527 (2016)

2. M. I. Love, W. Huber, S. Anders, Moderated estimation of fold change and dispersion for RNA-seq data with DESeq2. *Genome Biol.* **15**, 550 (2014).

3. D. W. Huang, B. T. Sherman, R. A. Lempicki, Systematic and integrative analysis of large gene lists using DAVID bioinformatics resources. *Nat. Protoc.* **4**, 44–57 (2009).

4. A. Dobin, et al., STAR: ultrafast universal RNA-seq aligner. *Bioinformatics* **29**, 15–21 (2013).

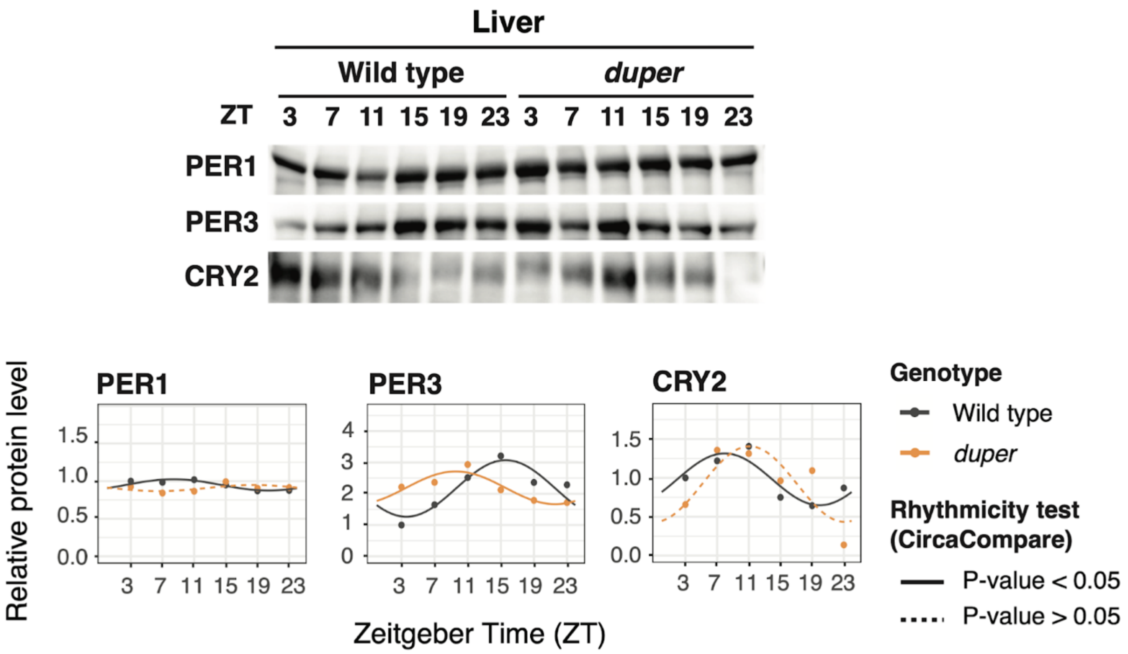

Fig. S1. Protein oscillation profiles of clock genes from the liver. Wild type and *duper* mutant tissue was collected at indicated times. Western blotting was performed on protein extracted from whole cell lysates with indicated antibodies. Normalized protein levels are shown in the plot. Significance of the rhythmicity test was performed using CircaCompare (Table S1), and represented with solid line ($p<0.05$) and dotted line ($p>0.05$). Wild types are colored in dark grey and *duper* are colored in orange.

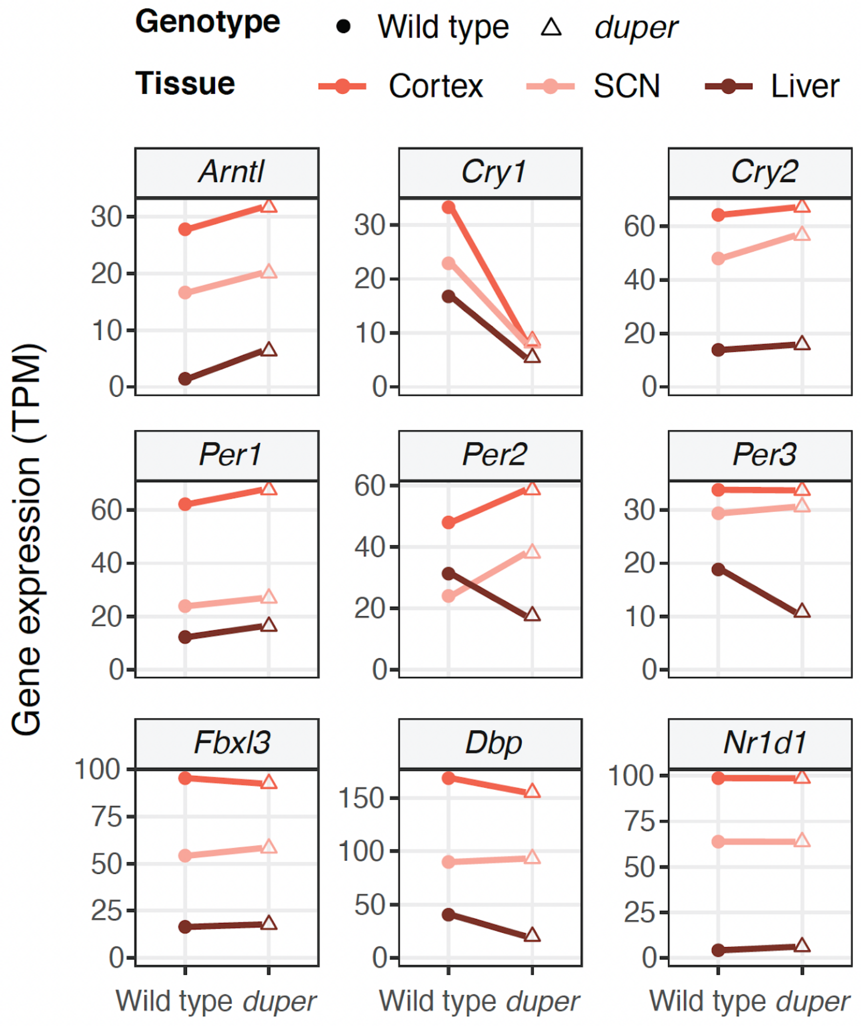

Fig. S2. Gene expression profiles of clock and clock related genes between wild type (LVG) and *duper* hamster at CT15 in cortex, SCN and liver. Wild types are represented in circles and *duper* are represented in triangles. The gradient of the segments indicate changes of gene expression (TPM) between the two genotypes.

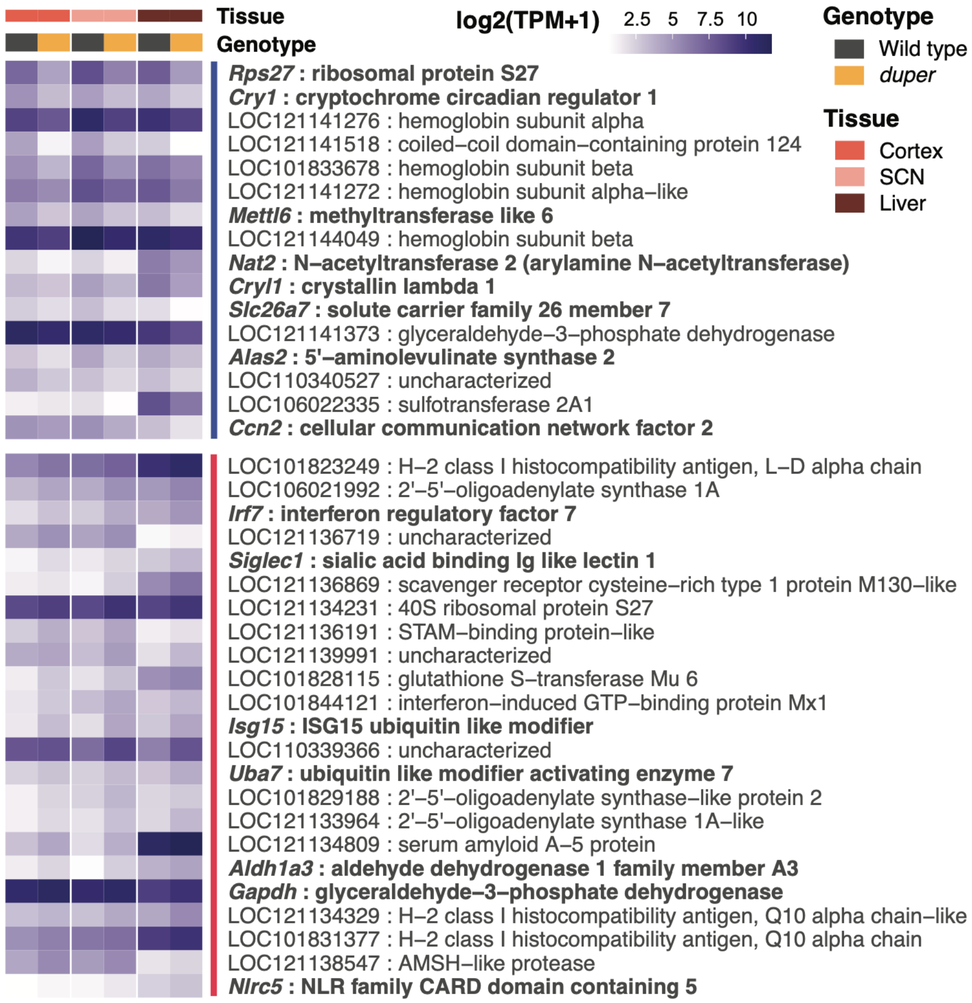

Fig. S3. Differentially expressed genes in *duper* in comparison to wild type (LVG) Syrian hamsters at CT15. Expression levels, log2(TPM+1), for genes that were differentially expressed (BH.Q <0.05 and log2 fold-change >0.58) between *duper* and wild type Syrian hamsters in cortex, SCN and liver are shown in the heatmap. Genes that were down- and up-regulated in *duper* are clustered in blue(top) and red(bottom), respectively. Genes in each cluster are ordered from top to bottom by their BH.Q value from DESeq2.

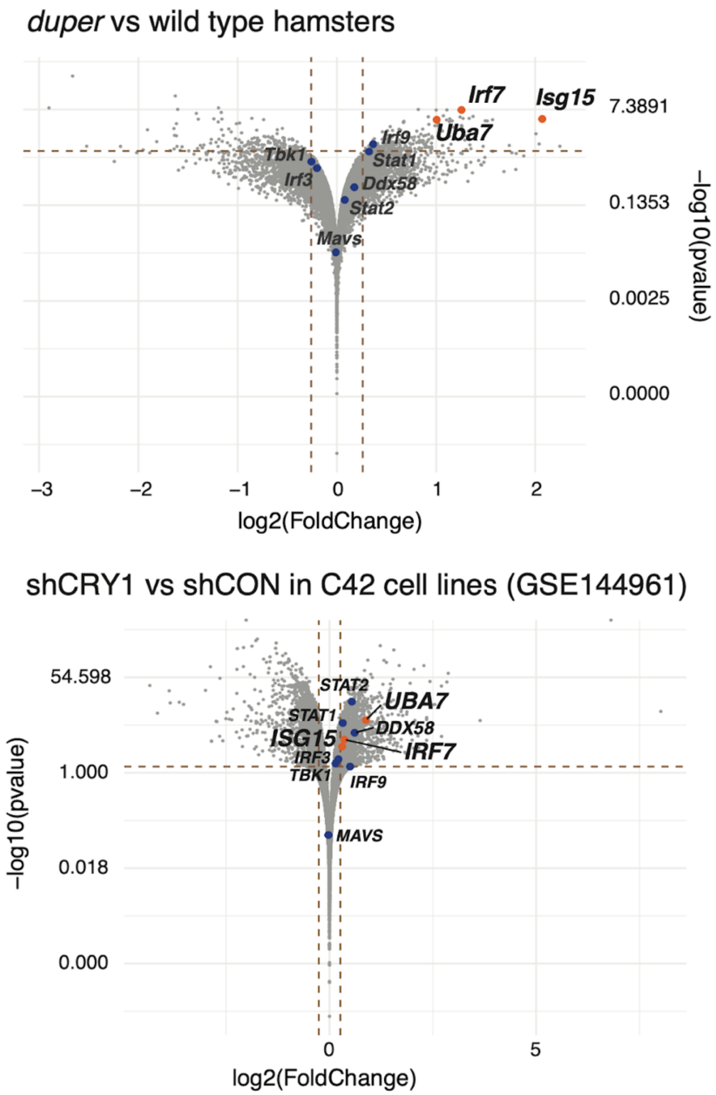

Fig. S4. Volcano plot of differential expressed genes(DEGs) from RNA-seq data. DEGs from *duper* vs wild type in hamsters (top) and *Cry1* shRNA (sh*Cry1)* knockdown vs control with shRNA (shCON) in C42 cell lines (bottom) are shown in the volcano plot. The 3 genes annotated to RIG-I-like receptor signaling pathway (*Isg15*, *Irf7* and *Uba7*) are labeled in orange and labeled with larger fonts. Other genes involved in the ISG15 conjugation pathway are labeled in blue and labeled with smaller fonts. Horizontal lines indicate p-value of 0.05; vertical lines indicate fold-change of 1.2.

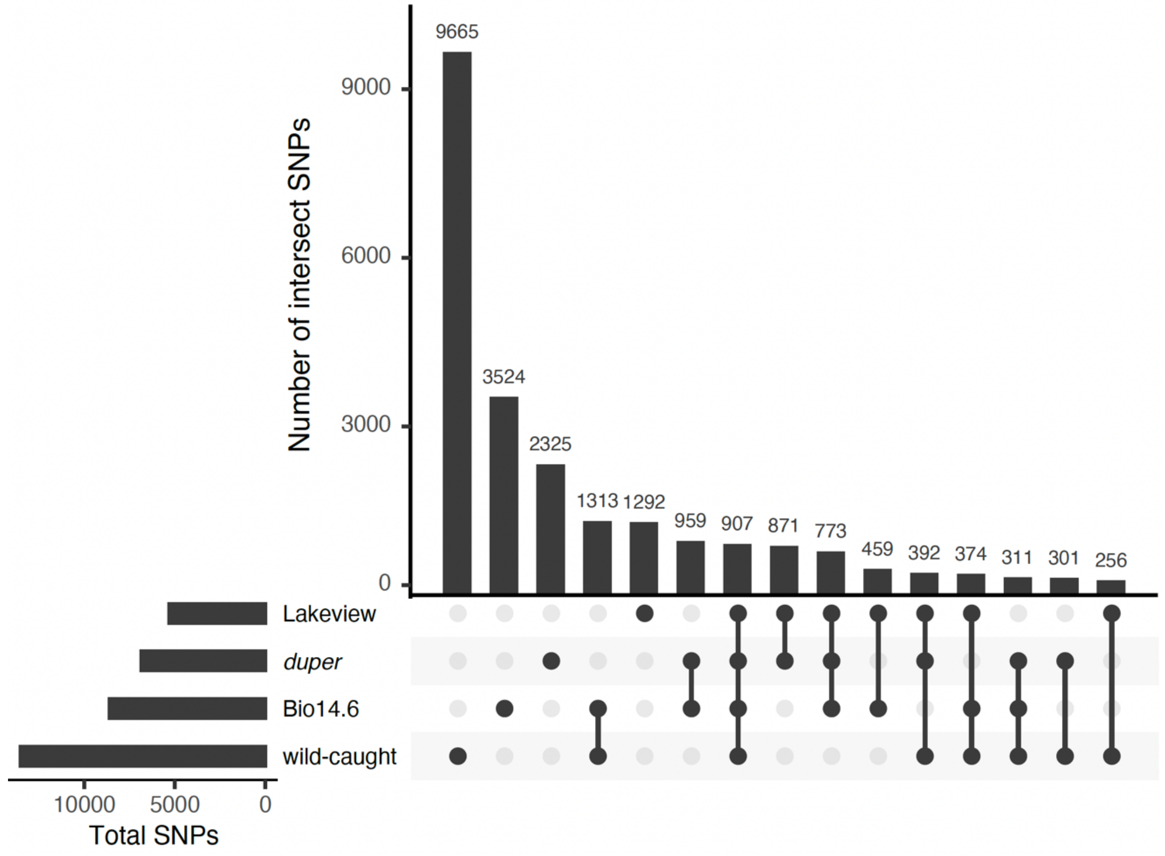

Fig. S5. SNPs map of Syrian hamsters at the coding regions. The UpSetR plots show number of intersect SNPs between Lakeview (wild type Syrian hamster obtained from Charles River Laboratories), *duper* (Syrian hamster from Lakeview ecotype but independently bred since the identification of *tau* mutations in 1988), Bio14.6 (wild type Syrian hamster obtained from Biobreeders), and wild-caught (descendants of wild-caught Syrian hamsters described in Weinert et al. 2001).

Table S1. Summary of rhythmicity test and comparison between *duper* and wild-type using CircaCompare.

	CRY2	PER1	PER3
WT.rhythmicity.pvalue	0.041	0.030	0.026
Duper.rhythmicity.pvalue	0.113*	0.231*	0.028
WT.mesor	0.981	0.950	2.169
Duper.mesor	0.946	0.908	2.210
mesor.diff.est	-0.035	-0.042	0.042
mesor.diff.pvalue	0.839	0.148	0.826
WT.amp	0.333	0.075	0.904
Duper.amp	0.459	0.046	0.526
amp.diff.est	0.126	-0.029	-0.378
amp.diff.pvalue	0.604	0.452	0.192
WT.peak	7.951	8.389	15.591
Duper.peak	11.926	19.343	10.328
phase.diff	3.976	10.954	-5.263
phase.diff.pvalue	0.140	0.004	0.013
shared.phase.est	24	24	24

* Time-series protein abundances do not pass the rhythmicity test (p-value > 0.05)

Dataset S1 (separate file). Differentially expressed genes in *duper* in comparison to wild type (LVG) Syrian hamsters at CT15.
